# Effects of Process Parameters on Forming Quality and Microstructure of FeCrAl-ODS Alloy Fabricated by Selective Laser Melting

**DOI:** 10.3390/ma18112462

**Published:** 2025-05-24

**Authors:** Shenghua Zhang, Fudong Li, Yu Wang, Hongwen Su, Jun Li

**Affiliations:** 1 School of Materials Science and Engineering, North University of China, Taiyuan 030051, China; 18298897601@163.com (F.L.); wangyu@nuc.edu.cn (Y.W.); 2 Shanxi Huachuang Laser Technology Co., Ltd., Changzhi 046000, China; sxszshw@163.com; 3 Taiyuan Iron and Steel (Group) Co., Ltd., Taiyuan 030051, China

**Keywords:** FeCrAl-ODS alloy, selective laser melting, process parameter, forming quality, microstructure

## Abstract

This study systematically investigated the effects of selective laser melting (SLM) process parameters on the forming quality and microstructure of FeCrAl oxide dispersion-strengthened (ODS) alloy. Through orthogonal experimental design, the influences of laser power (300–320 W), scanning speed (650–850 mm/s), and hatch spacing (0.05–0.07 mm) on the surface morphology and internal defects of as-built samples were analyzed. The microstructural evolution under different volumetric energy densities (VED) was also analyzed. The results indicate that hatch spacing significantly affected crack and pore formation, with minimal defects observed at 0.06 mm. Excessive laser power (320 W) or VED (318.0 J/mm^3^) led to elevated melt pool temperatures, causing element evaporation, grain coarsening, and <100> preferential oriented texture, thereby reducing hardness to 234 HV. The optimal parameters—laser power of 310 W, scanning speed of 650 mm/s, and hatch spacing of 0.06 mm (VED 265.0 J/mm^3^)—yielded the highest hardness (293 HV), fine-grained structures, and a high proportion of low-angle grain boundaries (LAGBs) with significant residual stress. This research provides a theoretical foundation for optimizing SLM processes for FeCrAl-ODS alloys.

## 1. Introduction

FeCrAl-ODS alloys exhibit exceptional performance in high-temperature environments, characterized by superior oxidation resistance, robust tolerance to irradiation, and enhanced mechanical properties, making them promising candidates for nuclear reactor cladding and high-temperature structural components [1,2]. However, traditional powder metallurgy methods for fabricating ODS alloys face limitations, including complex processing, high costs, and challenges in forming complex geometries [3,4]. Additionally, subsequent rolling processes often fail to eliminate elongated grain structures, leading to anisotropic properties [5]. To address these issues, emerging techniques such as additive manufacturing (AM) have been explored [6,7,8,9,10]. Among these, selective laser melting (SLM), an AM technology, enables the direct fabrication of high-precision complex parts through layer-by-layer melting. Its rapid cooling rates and micro-melt pool characteristics facilitate the uniform dispersion of nano-sized oxide particles and inhibit oxide particles’ coarsening [11]. Nevertheless, the influence of SLM process parameters on the microstructure and properties of FeCrAl-ODS alloys with high oxide content remains insufficiently studied.

Prior studies highlight volumetric energy density (VED) as a critical factor in controlling melt pool dynamics, defect formation, and grain structure during SLM [12,13]. For instance, Ahmed et al. [14] found that, when processing the crack-susceptible and unweldable high-temperature nickel-based alloy CM247LC, laser power and hatch spacing had minimal effects on crack formation. However, optimizing hatch spacing contributed to a significant reduction in cracking. Liu et al. [15] reported that, during the SLM processing of SUS316L, scanning speed had a major influence on porosity. Li et al. [16] observed that a high scanning speed or low laser power promoted the transition from columnar to equiaxed grains. Greco et al. [17] studied the effect of laser power under constant VED conditions, and found that increasing the laser power reduced surface roughness perpendicular to the build direction, while simultaneously increasing relative density and microhardness. Yang et al. [18] reported that excessive VED increases porosity and cracks in 316 L stainless steel, while Zhang et al. [19] demonstrated that nano-oxide particles suppress phase transformation via a pinning effect. However, research on the SLM process optimization and microstructural evolution of FeCrAl-ODS alloys, particularly those with high oxide content, is still limited. This study systematically investigates the effects of laser power, scanning speed, and hatch spacing on the forming quality and microstructure of an FeCrAl-ODS alloy containing 0.91 wt. % Y_2_O_3_. The higher addition of Y_2_O_3_ is intended to improve the mechanical property of FeCrAl-ODS in this study. Through metallographic analysis, electron backscatter diffraction (EBSD), and microhardness testing, the correlation between process parameters and mechanical performance is elucidated, providing theoretical guidance for the additive manufacturing of high-performance FeCrAl-ODS alloys.

## 2. Experimental Methods

**Figure 1:**
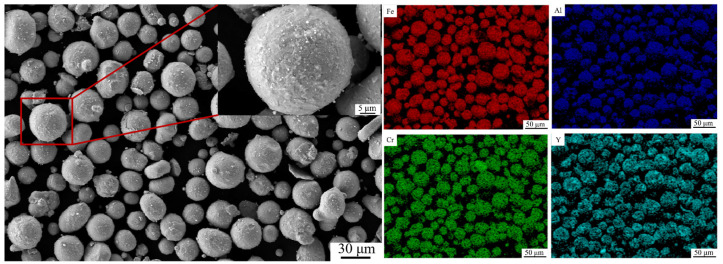
Second electron images and elemental mappings of FeCrAl-ODS alloy powder.

FeCrAl alloy powder with a particle size range of 15–53 μm and Y_2_O_3_ powder with a particle size of 30 nm were used in the experiment. The FeCrAl-ODS alloy powder with 1 wt. % yttria addition was prepared via low-energy ball milling. Stainless steel balls with diameters of 10 mm and 5 mm, blended in a 1:1 weight ratio, are used for ball milling. The milling parameters were as follows: a ball-to-powder ratio of 5:1, a milling duration of 4 h, and a rotational speed of 100 rotation per minute. To prevent powder contamination, the mixed powder was ball-milled under high-vacuum conditions (<0.1 Pa). Contamination originating from the stainless pot and balls is negligible under low-energy ball milling conditions. The morphology and surface elemental distribution of the as-milled powders were analyzed via a Zeiss Sigma 300 field-emission scanning electron microscope (SEM, Carl Zeiss AG, Oberkochen, Germany) integrated with energy-dispersive X-ray spectroscopy (EDS), as shown in Figure 1. The FeCrAl alloy powders maintained good sphericity after milling. A magnified view of a single powder particle within the red box in Figure 1 is shown in the inset, revealing that the nano-sized Y_2_O_3_ particles adhered to the surface of the FeCrAl alloy powders after milling. Elemental mappings indicate that the Y_2_O_3_ particles were relatively uniformly distributed on the powder surface. A small fraction of irregularly shaped particles was observed; however, the overall powder morphology meets the spreading requirements for SLM processing. The size of oxide particles in the as-built samples was 30–40 nm, and they distributed uniformly in the matrix, according to the prior study [11].

The cubic specimens (10 × 10 × 10 mm^3^) were fabricated via a commercial SLM system (EP-M150, E plus 3D Technology Co., Ltd, Beijing, China) equipped with a fiber laser (spot diameter: 70 µm) capable of achieving a maximum scanning speed of 8 m/s. To minimize anisotropic defects, a 67° interlayer rotation strategy was applied during scanning. The thickness L of the powder spread was 30 µm. The substrate made of 304 stainless steel was preheated to 120 °C prior to processing, and argon shielding gas was utilized to maintain an oxygen concentration below 0.1 vol.% within the build chamber throughout the forming stage. Based on previous studies [11], the printing parameter ranges were selected as follows: laser power (*P*) of 300–320 W, scanning speed (*v*) of 650–850 mm/s, and hatch spacing (*H*) of 0.05–0.07 mm. The Y content in the fabricated alloy samples was determined to be 0.70 wt. % using inductively coupled plasma optical emission spectroscopy (ICP-OES, Agilent 720ES, Agilent Technologies Inc., Santa Clara, CA, USA), corresponding to Y_2_O_3_ contents of 0.91 wt. %. The analysis of Y content was performed with the following parameters: RF power of 1.2 KW, plasma flow of 15 L/min, auxiliary flow of 1.5 L/min, nebulizer flow of 0.75 L/min, sample uptake delay of 15 s, and 3 replicates. An inverted metallurgical microscope (MDS 400) was employed to observe the morphology and distribution of defects in the mechanically ground and polished samples. Electrolytic polishing was conducted using a solution of 10% HClO_3_ and 90% C_2_H_5_OH, and electron backscatter diffraction (EBSD) was used to characterize the grain size and crystallographic texture of the specimens fabricated under different VED. The EBSD data were processed using Channel 5 software. The microhardness of each sample was measured 5 times using an HV-1000B Vickers (Laizhou Hengyi Testing Instrument Co., Ltd., Laizhou city, China) hardness tester under a load of 0.2 kgf, with a dwell time of 15 s.

## 3. Results and Discussion

### 3.1. Effect of Process Parameters on the Forming Quality

**Figure 2:**
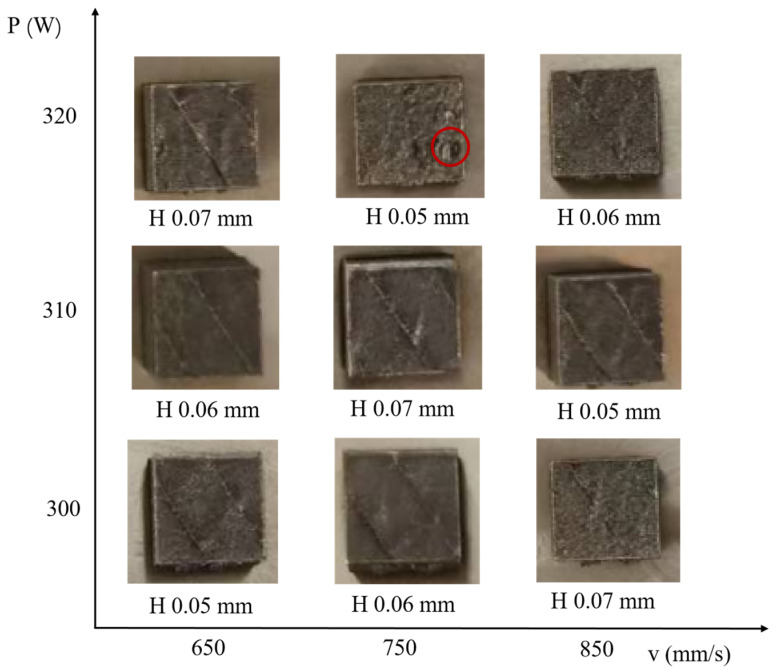
Photos of as-built samples.

A three-factor, three-level orthogonal experimental design was employed, as shown in Table 1. The VED was calculated using the formula VED = *P*/*vHL*. Top-view images of the fabricated samples are presented in Figure 2. Under the selected processing parameters, all specimens were successfully fabricated without any observable cracking on the surface. Notably, sample 8 exhibited a relatively rough surface with visible pits, as indicated by the red circles in Figure 2. This may be attributed to the high volumetric energy density resulting from the combination of high laser power and small hatch spacing during the scanning processes. Excessive energy input can lead to elevated melt pool temperatures, which may cause the evaporation of alloying elements or spattering of the powder [13]. As layers accumulate, such effects can result in uneven surface morphology. Interestingly, compared to sample 8, sample 1 had the same hatch spacing and a slower scanning speed, resulting in a higher corresponding volumetric energy density, yet no surface pits were observed on its top surface. This suggests that the high energy density caused by reduced scanning speed has a relatively minor impact on forming quality, whereas energy density increases due to elevated laser power have a more significant effect on surface integrity.

**Figure 3:**
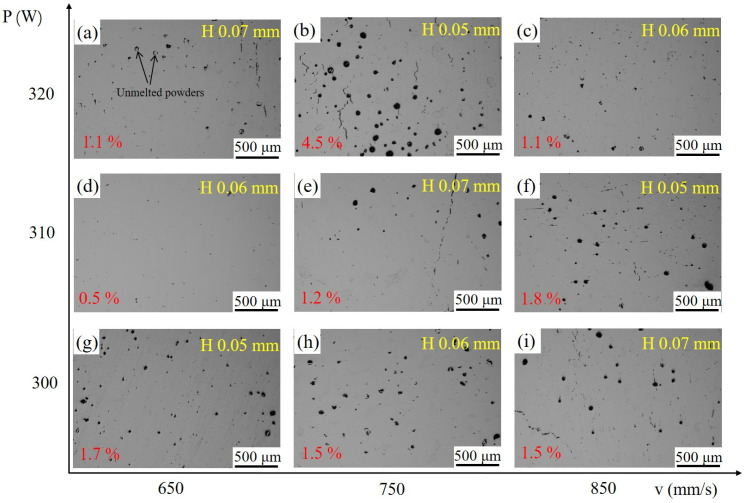
Metallographic micrographs of as-built samples. (**a**) P (320 W), v (650 mm/s), H (0.07 mm); (**b**) P (320 W), v (750 mm/s), H (0.05 mm), (**c**) P (320 W), v (850 mm/s), H (0.06 mm); (**d**) P (310 W), v (650 mm/s), H (0.06 mm), (**e**) P (310 W), v (750 mm/s), H (0.07 mm); (**f**) P (310 W), v (850 mm/s), H (0.05 mm), (**g**) P (300 W), v (650 mm/s), H (0.05 mm); (**h**) P (300 W), v (750 mm/s), H (0.06 mm), (**i**) P (300 W), v (850 mm/s), H (0.07 mm).

Metallographic images provide a more intuitive means of observing the types and distributions of internal defects in the as-built blocks. Figure 3 presents the metallographic micrographs of the nine printed samples. It can be observed that when the hatch spacing is 0.07 mm, a significant number of pores and extensive cracking are present. In contrast, when the hatch spacing is reduced to 0.06 mm, the number of cracks is significantly reduced. However, when the hatch spacing further decreases to 0.05 mm, cracks appear. This indicates that crack formation is closely related to hatch spacing. A larger hatch spacing results in a lower energy density, leading to poor overlap between adjacent melt tracks and the formation of more cracks. When the hatch spacing is too low, hot cracking susceptibility increases. Additionally, unmelted powder can also be observed, as shown in Figure 3a. The porosity percentages are quantitatively shown in red font at the bottom left of each figure. When the hatch spacing was 0.05 mm, more pores (>1.7%) were observed within the samples, particularly at a laser power of 320 W, where both the number and size of pores were the greatest, as shown in Figure 3b. These large, round pores are typically formed by the evaporation of light elements, indicating that a laser power of 320 W is excessive for a hatch spacing of 0.05 mm. When the hatch spacing was increased to 0.06 mm, the number of defects in the fabricated parts was significantly reduced. At a laser power of 310 W and a scanning speed of 650 mm/s, only a small number of fine pores (0.5%) were observed in the sample. Therefore, for the alloy system studied in this work, the optimal processing parameters are a laser power of 310 W, scanning speed of 650 mm/s, and hatch spacing of 0.06 mm.

### 3.2. Effect of Process Parameters on the Evolution of Microstructure

**Figure 4:**
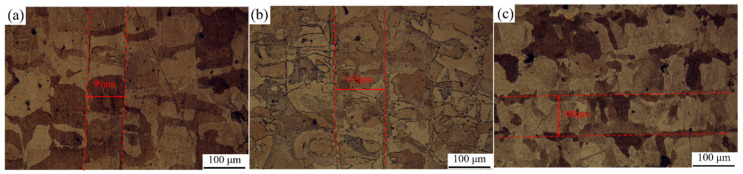
Metallographic micrographs of samples’ cross-sections with different VEDs—(**a**) 202.6 J/mm^3^, (**b**) 265.0 J/mm^3^, (**c**) 318.0 J/mm^3^.

In order to obtain a clear evolution of the microstructure with VED variation, three samples with around 60 J/mm^3^ increments were designed. The *P* was set as constant to reduce the complications associated with different process parameters. To investigate the effect of VED on the microstructure of SLM-fabricated alloys, a low VED of 202.6 J/mm^3^ and a high VED of 318.0 J/mm^3^ were selected based on the optimal processing condition, which corresponded to a VED of 265.0 J/mm^3^. The specific processing parameters are listed in Table 2. Metallographic images of the cross-sections of the etched samples are shown in Figure 4. After etching, distinct melt pool tracks could be observed in all three samples. As the VED increased due to a reduction in scanning speed, the width of the melt pools significantly increased, and columnar grains aligned parallel to the laser scanning direction appeared at the melt track boundaries, as shown in Figure 4a,b. In contrast, when VED increased due to a decrease in hatch spacing, the melt pool width became narrower, as observed in Figure 4b,c. This indicates that the melt pool width is influenced by both the heat input and the hatch spacing. Additionally, grain coarsening was observed at the melt pool boundaries with increasing VED.

**Figure 5:**
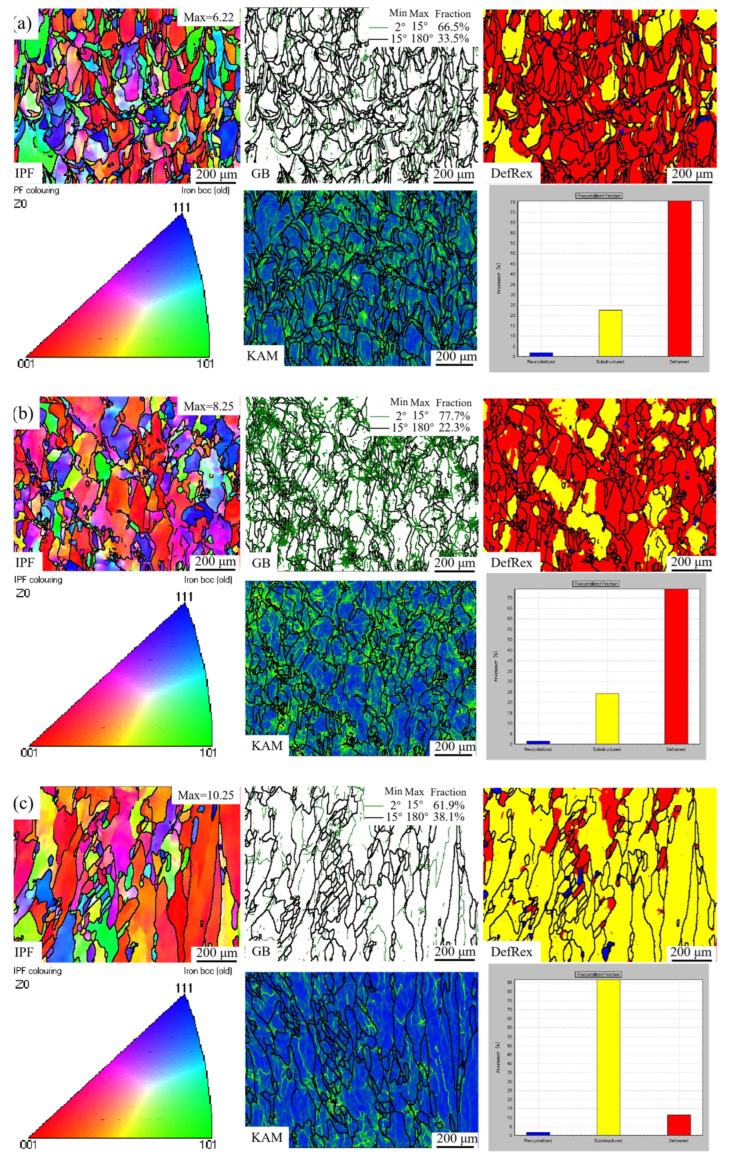
IPF, GB maps, DefRex maps, IPF coloring, KAM maps, and the recrystallized fraction of samples with different VEDs—(**a**) 202.6 J/mm^3^, (**b**) 265.0 J/mm^3^, (**c**) 318.0 J/mm^3^.

Figure 5 presents the inverse pole figures (IPF), grain boundary (GB) maps, deformed–recrystallized (DefRex) maps, and kernel average misorientation (KAM) maps of samples printed under different VED conditions. With increasing VED, the proportion of red-colored grains in the IPF gradually increased, and the maximum texture intensity of the samples became more pronounced. This indicates an enhanced preferential orientation along the <100> direction with increasing heat input. This phenomenon is attributed to the elevated melt pool temperature and reduced cooling rate at higher VED levels, which allow a larger portion of the solidified section to reach recrystallization temperatures. As a result, a pronounced <100> preferential orientation formed in the cubic structured alloy. Consequently, excessively high VED may induce anisotropy in the mechanical properties of SLM-fabricated samples, potentially limiting their practical applications. Most of the grains in the three samples exhibit columnar morphology. When the VED exceeds 300 J/mm^3^, the excessively high melt pool temperature leads to grain coarsening and the abnormal growth of some substructured grains.

In the GB maps, green lines represent LAGBs, while black lines indicate high-angle grain boundaries (HAGBs). As VED increased, the proportion of LAGBs initially rose and then decreases, a trend that is also reflected in the corresponding KAM maps, where the internal strain follows a similar pattern. This is because, in general, a higher proportion of LAGBs indicates a higher dislocation density and greater internal stress. Due to the extremely high cooling rate during the SLM process (10^6^–10^7^ K/s) [12], the solidified grains were predominantly deformed grains. At a VED of 202.6 J/mm^3^, the melt pool temperature was relatively low, resulting in smaller grain sizes and moderate levels of LAGBs and internal strain. The heat-affected zone adjacent to the melt pool was narrow, and most of the microstructure consisted of deformed grains. When the VED increased to 265.0 J/mm^3^, the melt pool temperature rose. However, due to the pinning effect of the nano-sized oxide particles, grain growth was effectively suppressed, and the transition from deformed grains to substructured grains was inhibited. This led to the accumulation of internal stress and an increased fraction of LAGBs, as the stored energy from rapid solidification was not released. At a higher VED of 318.0 J/mm^3^, the proportion of HAGBs increased sharply, accompanied by a significant decrease in internal strain, as shown in the KAM maps. This may be attributed to the enhanced thermal driving force at elevated temperatures overcoming the pinning effect of the oxide particles, thereby promoting the transformation of a large number of deformed grains into substructured grains.

### 3.3. Effect of VED on the Hardness of FeCrAl-ODS Alloys

**Figure 6:**
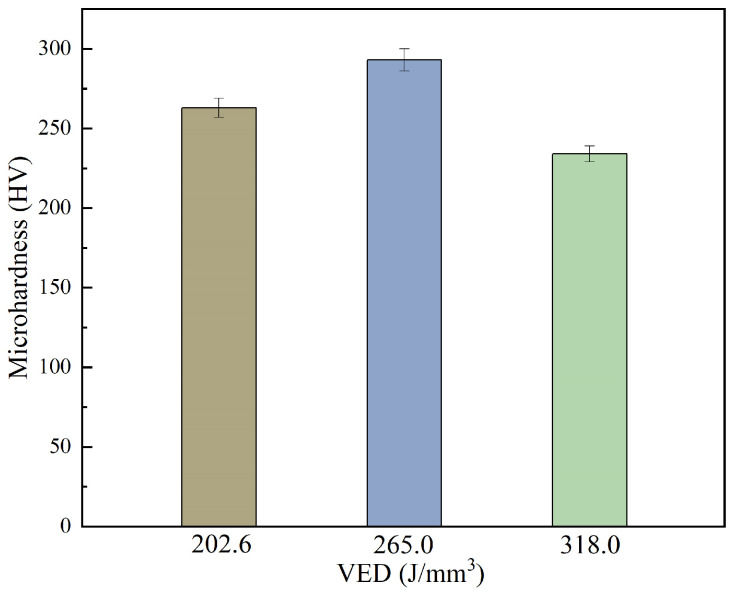
Microhardness comparison of samples with different VEDs.

During the SLM process, the VED directly determines the cooling rate, melt pool size, and thermal cycling frequency of the alloy material, thereby influencing the evolution of the microstructure and ultimately leading to differences in mechanical properties. The hardness of the samples fabricated under three different VED conditions was measured, and the comparison is shown in Figure 6. Owing to its finer grain size, high dislocation density, and significant internal stress, the sample fabricated at a VED of 265.0 J/mm^3^ exhibited the highest hardness, reaching 293 HV. At a lower VED of 202.6 J/mm^3^, the reduced internal stress and dislocation density resulted in a decreased hardness of approximately 263 HV. In contrast, at a high VED of 318.0 J/mm^3^, grain coarsening and the release of internal stress led to the lowest hardness value, around 230 HV. Therefore, the hardness of SLM-fabricated FeCrAl-ODS alloys is closely correlated with the microstructural evolution induced by the scanning parameters. An appropriate VED can optimize the alloy hardness by controlling grain size, the fraction of substructured grains, and internal stress. The hardness values of FeCrAl-ODS alloys with 0.1 wt. % Y_2_O_3_ addition fabricated by SLM and conventional FrCrAl-ODS were 275 HV and around 300 HV [11,20]. This means that the hardness increases with an increase in Y_2_O_3_ addition for printed FeCrAl-ODS alloys, and their mechanical properties are comparable with those of the FeCrAl-ODS alloys fabricated via powder metallurgy methods. It is expected that a proper post-process heat treatment could further achieve a balance between strength and ductility in the alloy.

## 4. Conclusions

By optimizing SLM process parameters, FeCrAl-ODS alloys with high forming quality and superior hardness were successfully fabricated. The evolution of the microstructure under varying VED values was investigated, and the following conclusions were drawn:

(1) Optimization of forming quality—Hatch spacing critically influenced defect distribution. At 0.06 mm, overlapping melt tracks minimized cracks and pores, whereas excessive laser power (320 W) or VED (318.0 J/mm^3^) caused surface pits due to element evaporation from overheating. The scanning speed has a relatively minor impact on forming quality;
(2) Microstructural regulation—VED significantly affected grain structure and texture. At 265.0 J/mm^3^, nano-sized Y_2_O_3_ particles inhibited grain coarsening via pinning effects, forming fine grains with a high fraction of LAGBs and achieving a peak hardness of 293 HV. Conversely, excessive VED (318.0 J/mm^3^) induced abnormal grain growth and released internal stress, reducing hardness to 230 HV. A moderate VED is conducive to control microstructure evolution;
(3) Optimal parameters—The combination of 310 W laser power, 650 mm/s scanning speed, and 0.06 mm hatch spacing (VED 265.0 J/mm^3^) balanced forming quality and mechanical performance, laying a foundation for engineering applications of FeCrAl-ODS alloys.

Future work should focus on optimizing strength–ductility trade-offs through post-processing heat treatments and exploring residual stress control mechanisms during complex component fabrication.

## Figures and Tables

**Table 1:** SLM process parameters of orthogonal experimental design.

Sample ID	*P* (W)	*v* (mm/s)	*H* (mm)	VED (J/mm^3^)
1	300	650	0.05	307.7
2	300	750	0.06	222.2
3	300	850	0.07	168.1
4	310	650	0.06	265.0
5	310	750	0.07	196.8
6	310	850	0.05	243.1
7	320	650	0.07	234.4
8	320	750	0.05	284.4
9	320	850	0.06	209.2

**Table 2:** Specific SLM process parameters of samples with different VED.

Sample ID	*P* (W)	*v* (mm/s)	*H* (mm)	VED (J/mm^3^)
1	310	850	0.06	202.6
2	310	650	0.06	265.0
3	310	650	0.05	318.0

## Data Availability

The original contributions presented in this study are included in the article. Further inquiries can be directed to the corresponding authors.
